# A twist in the tail

**DOI:** 10.7554/eLife.02386

**Published:** 2014-03-04

**Authors:** Karen Guillemin, Annah S Rolig

**Affiliations:** 1 **Karen Guillemin** is at the Institute of Molecular Biology, University of Oregon, Eugene, United Statesguillemin@molbio.uoregon.edu; 2 **Annah S Rolig** is at the Institute of Molecular Biology, University of Oregon, Eugene, United States

**Keywords:** Vibrio, fischeri, cholerae, flagella, LPS, animal-microbe interactions, other

## Abstract

Lipopolysaccharide molecules released by the bacteria *Vibrio fischeri* when it rotates its flagella prompts its host, the Hawaiian bobtail squid, to prepare for its arrival.

**Related research article** Brennan CA, Hunt JR, Kremer N, Krasity BC, Apicella MA, McFall-Ngai MJ, Ruby EG. 2014. A model symbiosis reveals a role for sheathed-flagellum rotation in the release of immunogenic lipopolysaccharide. *eLife* **3**:e01579. doi: 10.7554/eLife.01579

**Image** The Hawaiian bobtail squid houses bioluminescent bacteria in its own body (Credit: Jamie Foster)

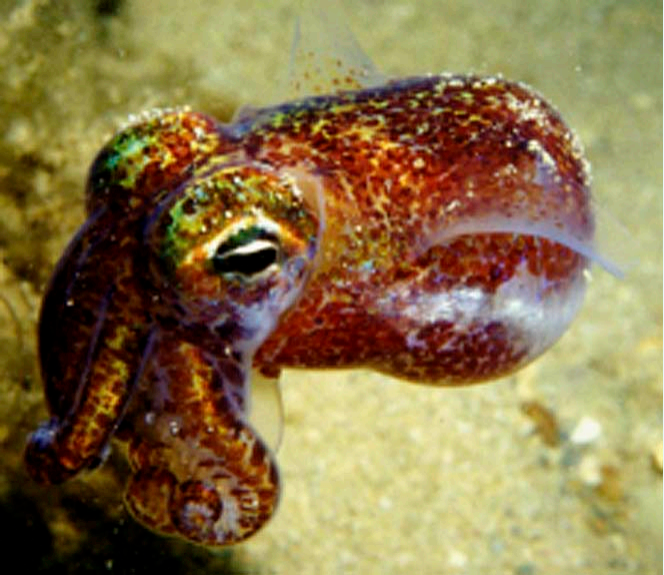

All animals live with microbes on and inside their bodies—within humans, microbes can outnumber cells by a factor of ten—and animals engage with these microbes via the mutual exchange of different molecules. Animal-microbe interactions are often thought of as adversarial, with the host valiantly defending itself against the nefarious pathogens which, in turn, employ a range of strategies to evade the immune system of their host. However, most of the microbes that live with animals are harmless, and many animal–microbe interactions can offer mutual benefits to both parties.

Many bacteria have tail-like structures called flagella that they rotate in order to move around. A flagellum is made up of many proteins called flagellins, which are continuously shed and can alert the immune system to the presence of a bacterial pathogen. The flagella of some pathogens are covered by a sheath, and it has been generally assumed that the purpose of this sheath is to prevent the telltale flagellin proteins being detected by the host (Geis et al., 1993). Now, in *eLife*, Edward Ruby and colleagues at the University of Wisconsin–Madison and the University of Iowa—including Caitlin Brennan as first author—turn this story on its head and show that some microbes rely on this sheath to communicate with their host (Brennan et al., 2014).

The bacterial protagonist in this story is *Vibrio fischeri*, a bioluminescent bacterium that colonizes newly hatched Hawaiian bobtail squids (Nyholm and McFall-Ngai, 2004). When the squid detects the presence of the *V. fischeri* bacteria, it makes major changes to its ‘light organ’ to accommodate the bacteria. This interaction is ‘mutualistic’ rather than adversarial because both parties benefit. The squid uses the light emitted by the bacteria to obscure its silhouette during its nocturnal wanderings of the moonlit ocean, which helps it avoid being spotted by predators. In return, the squid provides the bacteria with sugars and other nutrients. Ruby and co-workers show that the bacteria make their presence known by rotating their sheathed flagella, which results in increased shedding of a molecule called lipopolysaccharide, or LPS for short (Brennan et al., 2014).

Flagella help bacterial pathogens to infect their hosts, mainly because they allow the bacteria to move through host tissues, to locate preferred sites to colonize, and to stick to specific host cells. Interestingly, several important pathogens—including *Vibrio cholerae*, which causes cholera, and *Helicobacter pylori*, which can cause stomach ulcers—coat their flagella with a sheath derived from their outer membrane. However, investigating the function of this sheath has been hindered by the lack of mutants that do not have sheaths, and by the difficulty of separating the flagella’s role in moving the bacteria from other possible functions.

To explore the role of the sheathed flagella, Brennan et al. took advantage of the tightly choreographed sequence of interactions between *V. fischeri* and its squid host. Previously, work from Ruby’s lab had demonstrated that flagellar-based motility is required for *V. fischeri* to colonize the light organ to sufficient levels to generate light (Graf et al., 1994). However, the squid can detect the *V. fischeri* bacteria before they start their journey into the light organ (Kremer et al., 2013), and the host responds to these early colonizers by instructing specific cells to commit suicide via a process called apoptosis (Foster et al., 2000).

Brennan et al. have now examined the ability of non-motile *V. fischeri* mutants—that either lack flagella, or lack the ability to rotate their flagella—to signal to their squid hosts. Although these strains were present in the light organ, they did not trigger normal levels of apoptosis in the squid. This suggests that the rotation of the sheathed flagella is required to notify the host of *V. fischeri’s* arrival.

The normal apoptosis response in the squid, which happens early in the colonization of the light organ, is triggered by a molecule called ‘lipid A’ (Foster et al., 2000). Lipid A is a component of LPS and also stimulates the immune systems of animals. Brennan et al. hypothesized that rotating the sheathed flagellum sheds LPS, thereby producing adequate amounts of this signal to be perceived by the host (Figure 1). Consistent with this hypothesis, they found that the mutant strains released much less LPS than wild-type *V. fischeri*. Moreover, they demonstrated that the human pathogen *V. cholerae* also releases much less LPS when their flagella cannot rotate*.* However, *E. coli*, which do not have sheathed flagella, continue to release of LPS (albeit at low levels) even if their flagella cannot rotate (Brennan et al., 2014)*.*

**Figure 1.**
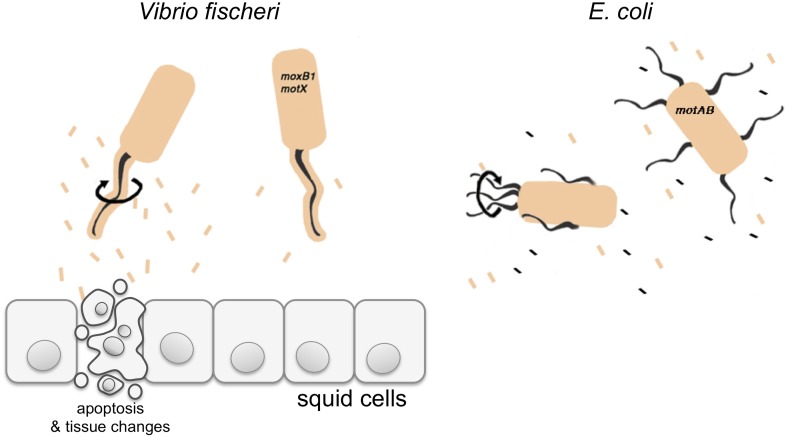
*Vibrio fischeri* and the Hawaiian bobtail squid. *V. fischeri* is one of a few bacterial species that cover their flagella in a sheath derived from their outer membrane (shown in beige). Previously it was thought that this sheath only stopped a protein called flagellin being shed from the flagellum, and thus prevented this protein being detected by the immune system of the host. Brennan et al. have now redefined the function of the flagella sheath by showing that rotation of *V. fischeri’s* sheathed flagella (depicted by the black arrow) induces the release of lipopolysaccharide (LPS, beige rectangles), and that LPS is required to trigger apoptosis and other changes in the squid to accommodate the bacteria. *V. fischeri* mutants whose flagella do not rotate (*motB1* and *motX*) release significantly less LPS and do not induce apoptosis. Many other bacteria, such as *E. coli,* have unsheathed flagella that shed flagellin monomers (black rectangles); however, both wild-type *E. coli* and mutants that cannot rotate their flagella (*motAB*) shed similar low levels of LPS.

Brennan et al. suggest that the apoptosis that promotes the normal development of the light organ is triggered by the LPS that it released when the bacteria rotate their sheathed flagella. It is interesting to speculate that concealing one molecule that stimulates the immune system, flagellin, with another, LPS, allows *V. fischeri* to manage its interaction with its host. Controlling its interaction with the host immune system is also important for the stomach pathogen *H. pylori,* which may wish to remain hidden*. H. pylori* have unusually structured LPS that trigger a muted immune response, a trait they may have evolved as a strategy for living with rotating, sheathed flagella that shed copious amounts of LPS (Ogawa et al., 1997).

A lesson from the work of Brennan et al. is that we should avoid typecasting bacteria and the different signals that they produce. For example, a building block of peptidoglycan—a component of the cell wall in bacteria—is known as ‘trachea toxin’ from studies of the whooping cough pathogen. However, the same molecule serves as another potent signal to trigger tissue changes in the squid-*Vibrio* system (Koropatnick et al., 2004). With the addition of the work by Brennan et al., we are reminded that the context in which the host interprets these powerful immune system-stimulating molecules likely determines the outcome of the interaction. Thus, moving forward, it will be interesting to learn the nature of the LPS structures that are shed from the sheathed flagellum, and how and why they are detected by the host to signal a desirable interaction, as opposed to an unwanted invasion.
